# The Perceived Likelihood of Outcome of Critical Care Patients and Its Impact on Triage Decisions: A Case-Based Survey of Intensivists and Internists in a Canadian, Quaternary Care Hospital Network

**DOI:** 10.1371/journal.pone.0149196

**Published:** 2016-02-12

**Authors:** Joseph Dahine, Louay Mardini, Dev Jayaraman

**Affiliations:** 1 Department of Critical Care, Royal Victoria Hospital, McGill University Health Center, Montreal, Quebec, Canada; 2 Department of Critical Care, Montreal General Hospital, McGill University Health Center, Montreal, Quebec, Canada; 3 Department of General Internal Medicine, Montreal General Hospital, McGill University Health Center, Montreal, Quebec, Canada; 4 Department of Critical Care, Jewish General Hospital, Montreal, Quebec, Canada; Azienda Ospedaliero-Universitaria Careggi, ITALY

## Abstract

**Introduction:**

There is high variability amongst physicians’ assessments of appropriate ICU admissions, which may be based on potential assessments of benefit. We aimed to examine whether opinions over benefit of ICU admissions of critically ill medical inpatients differed based on physician specialty, namely intensivists and internists.

**Materials and Methods:**

We carried out an anonymous, web-based questionnaire survey containing 5 typical ICU cases to all ICU physicians regardless of their base specialty as well as to all internists in 3 large teaching hospitals. For each case, we asked the participants to determine if the patient was an appropriate ICU admission and to assess different parameters (e.g. baseline function, likelihood of survival to ICU discharge, etc.). Agreement was measured using kappa values.

**Results:**

21 intensivists and 22 internists filled out the survey (response rate = 87.5% and 35% respectively). Predictions of likelihood of survival to ICU admission, hospital discharge and return to baseline were not significantly different between the two groups. However, agreement between individuals within each group was only slight to fair (kappa range = 0.09–0.22). There was no statistically significant difference in predicting ICU survival and prediction of survival to hospital discharge between both groups. The accuracy with which physicians predicted actual outcomes ranged between 35% and 100% and did not significantly differ between the two groups. A greater proportion of internists favoured non resuscitative measures (24.6% of intensivists and 46.9% internists [p = 0.002]).

**Conclusion:**

In a case-based survey, physician specialty base did not affect assessments of ICU admission benefit or accuracy in outcome prediction, but resulted in a statistically significant difference in level of care assignments. Of note, significant disagreement amongst individuals in each group was found.

## Introduction

Important decisions with regards to critical care admission as well as treatment options are made taking account not only patient preferences, but also physicians’ assessment of benefit [1, 2]. Patients who are denied ICU admission have higher mortality [2, 3]. However, predicting outcomes in critically ill patients is a complicated task, and health care professionals’ estimate of survival and quality of life of critically ill patients in general has been modest [4–9]. Twenty years ago, Cook and al. demonstrated a high variability amongst intensivists’ perceptions of what “appropriate” interventions entailed in a large Canadian survey of hypothetical cases [10]. More recently, physician-related variability in decision-making has been identified as a practical and ethical problem in highly value-laden and contentious areas such as in end of life decisions in the ICU [11–13], warranting strategies to reduce this variability such as process guidelines and improving awareness of one’s own practice [14]. Although end of life decisions have heavy implications for patients and resources, we believe that another critical point of potential variability occurs at triage. It has become apparent from studies performed during highly stressed resource-scarce environments, such as pandemics, that heterogeneity of practice can lead to mistrust and ethical issues between care providers and care receivers [15]. Consequently, identifying all possible factors contributing to heterogeneity is important. Hence, we sought to determine whether physician specialty base is one such factor. In our institutions, when hospitalised medical patients become critically ill, an ICU consult is initiated by the attending internist, if deemed appropriate. The intensivist will then decide on whether the admission is warranted. This assessment can, not infrequently, lead to disagreements between the requesting internist and assessing intensivist. As there may be systematic differences between these two groups based on their subspecialty choice [16, 17], we built a survey, based on five remote actual cases, probing various elements such as the appropriateness to ICU admission, probability of ICU survival and hospital discharge. The primary goal of the survey was to evaluate the variability amongst the physicians on these parameters, and to assess whether there are significant systematic differences between internists and intensivists as a group. Secondarily, we sought to compare the ability of each group predict actual outcomes (ICU survival and hospital discharge) and their views on appropriate levels of care for the patients.

## Methods

Fifteen cases were randomly chosen from all admissions to two adult ICUs from the medical floors between 2007 and 2009 within the McGill University network. Of the 15 cases, 5 cases that reflected 5 different, but common pathologies seen in an ICU setting were selected. Relevant information prior to the ICU admission was abstracted from the chart to build a vignette for each case. Chart review and conduct of this study were approved by the ethics committee of each institution. We then built a questionnaire that aimed to dichotomize answers. For each case, we asked participants to assess 8 different parameters including baseline function, likelihood of ICU and hospital survival, likelihood of return to baseline function, appropriateness of transfer to ICU, appropriateness of reason for referral, as well as the appropriate level of care (S1 Appendix) for the case patient. Answers were to be provided as “yes” or “no” (except for level of care assessment). We guided the participants to answer “yes” if they felt more than 70% certain to avoid moderacy bias. We piloted the questionnaire using an additional case not included in final survey to a sample of three attending physicians from the two groups. Comments from these individuals were submitted to a focus group and integrated to design the final questionnaire.

The survey was sent electronically to all internists who round on the medical wards and practicing intensivists at the three adult sites in our network through the One45 system. One45 is a web-based platform that encompasses a database of all faculty and students registered at our Medicine Faculty and/or practicing at our university hospital network. Registration is compulsory and automatically done upon registration to the Faculty. The platform allows for multiple capabilities such as filling evaluations and surveys. It then allows data collection anonymously. Participants were not aware the cases were real. Intensivists who round on both the ICU and medical wards were considered as intensivists for the analysis.

Concordance within groups was performed using kappa coefficient. Degree of agreement based on the kappa coefficient was assessed as described previously [18–21]. Test for proportions and Wilcoxon rank sum tests were used as appropriate for comparisons between groups. All significant testing were two tailed with a significance set at 0.05. Analysis was performed using STATA (version 10).

## Results

Twenty-two internists and twenty-one intensivists completed the survey with a response rate of 34.9% and 87.5% respectively. Further respondants’ characteristics are found in Table 1. Of note, 10 of the 21 intensivists also rounded on the medical wards. After combining the results from all five cases, we compared the average response of the internists to the intensivists for every parameter assessed (Table 2). We found no statistically significant difference between the average responses of the two groups for any of the 7 parameters assessed. In contrast, when analyzing the agreement within each group, there was only slight agreement among the intensivists and the internists (Table 3) for all parameters. Agreement amongst intensivists for prediction of ICU survival and survival at hospital discharge was slight to fair with kappa values of 0.15 and 0.22, respectively, while amongst internists it was slight with kappa values of 0.09 and 0.16, respectively. The agreement amongst intensivists and internists at assessing patients’ baseline function was poor (kappa = 0.03 and 0.008, respectively). Similar results were obtained for assessment of appropriateness of consult and ICU admission (Table 3).

**Table 1:** Demographic Data.

	Intensivists	Internists
**Sex—% Female (SE)**	15% (3.6%)	60% (5.7%)
**Mean age—years (SD)**	47.9 (9.67)	47 (10.2)
Response rate (N)*	87.5% (21)	35% (22)

SD: Standard deviation; SE: Standard error

* Individuals who have mixed practices of internal medicine and intensive care were analyzed as intensivists

**Table 2:** Aggregate responses of Internists and Intensivists to the case scenarios.

Question	Intensivists -% yes (SE)	Internists -% yes (SE)	P value
Likelihood of survival to ICU	48% (4.7%)	49% (5.0%)	0.91
Likelihood of survival to hospital discharge	33% (4.4%)	31% (4.6%)	0.79
Likelihood of return to baseline	25% (2.5%)	25% (4.4%)	0.97
Different reason for consult?	31% (4.3%)	21% (4.2%)	0.11
Would you have asked for the consult if on ward?	61% (4.6%)	62% (5.0%)	0.87
Would you have accepted patient if in ICU?	77% (4.0%)	74% (4.6%)	0.61
Good functional baseline?	41% (4.7%)	41% (5.0%)	0.97

SE: Standard Error

**Table 3:** Agreement amongst intensivists and internists.

Question	Kappa Intensivists	Kappa Internists
Likelihood of survival to ICU	0.15	0.09
Likelihood of suvival to discharge	0.22	0.16
Likelihood of return to baseline	0.16	0.10
Different reason for consult?	0.12	0.05
Would you have asked for the consult if on ward?	0.21	0.28
Would you have accepted patient if in ICU?	0.02	0.07
Good functional baseline?	0.03	0.008

We compared each group’s estimation of ICU survival and survival at discharge with actual patient outcomes for all five cases (Table 4). The accuracy of predictions was similar between internists and intensivists ranging from as low as 35% to as high as 100% depending on the case.

**Table 4:** Accuracy of predictions (as compared with actual survey cases outcomes).

	Likelihood of survival to ICU—% correct predictions (SE)			Likelihood of survival to hospital discharge—% correct predictions (SE)		
	Intensivists	Internists	P value	Intensivists	Internists	P value
Case 1	78% (10.5%)	55% (10.1%)	0.11	70% (10.5%)	45% (10.9%)	0.11
Case 2	78% (9.2%)	85% (7.5%)	0.58	87% (7.8%)	100% (0%)	0.10
Case 3	74% (9.5%)	55% (10.9%)	0.20	96% (4.8%)	80% (8.4%)	0.11
Case 4	45% (11.5%)	35% (10.1%)	0.50	71% (11.0%)	70% (9.7%)	0.92
Case 5	39% (10.8%)	35% (10.5%)	0.78	52% (11.2%)	40% (10.9%)	0.43
Overall	61% (4.0%)	53% (4.4%)	0.30	75% (4.7%)	67% (3.4%)	0.25

SE: Standard Error

**Fig 1:**
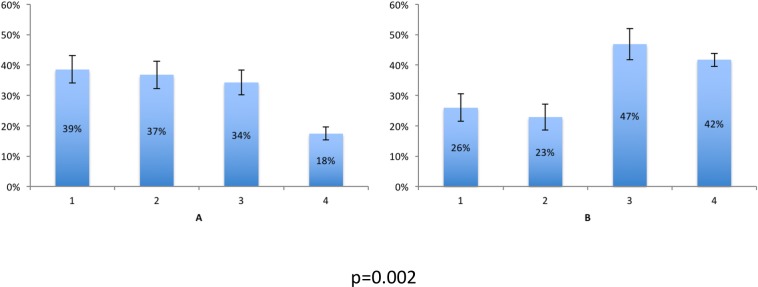
Aggregates of levels of care selected by respondents as appropriate for all presented cases. 1: Full care including resuscitative measures. 2: Full care including resuscitative measures but with exceptions (specified individually). 3: Maximum care excluding resuscitation and transfer to critical care units 4: Comfort care. A: Results for intensivists B. Results for internists

We examined the level of care distribution within each group. The intensivists group appeared to assign more aggressive level of care as 70% of the assigned levels of care were deemed appropriate for ICU (levels 1 or 2) (Fig 1A). In contrast, within the internists group, 50% of the assigned levels of care were not deemed appropriate for ICU (levels 3 or 4; p = 0.02) (Fig 1B).

The results did not change significantly from any of the above when intensivists who had a mixed practice were removed from data analysis.

## Discussion

Our results suggest that outcome prediction was similar between internists and intensivists, and the accuracy varied from case to case for both groups. Furthermore, we did not find any differences between the groups with regards to functional status, likelihood of ICU survival and hospital discharge as well as appropriateness of the consult or ICU admission. However, the variability between individuals is quite high as reflected by the poor concordance between individuals within each group. Cook and al, showed in a cross Canada survey large variability among critical care professionals concerning level of intervention and aggressiveness of treatment [10]; and, this variability could be explained in part by the personal values of the health care providers. Almost 20 years later, our study shows that there is still wide variability not only among critical care physicians, but also among internists. Thus disagreements between the requesting internists and the assessing intensivists are possibly due to individual differences (e.g. set of values, past experiences, etc.) rather than systematic differences between the two groups (e.g. their training background, their scope of practice, etc.). Further work with a larger cohort would be necessary to study which individual factors contribute to this variability.

Interestingly, the one difference between internists and intensivists was in the assessment of the appropriate level of intervention, with intensivists preferring more aggressive levels of intervention. Previous studies have demonstrated that physicians attribute levels of care to patients based on perceived quality of life [22]. However we did not find any differences between the groups in terms of their assessment of functional status or prognosis. It is possible that there are other factors such as a more aggressive outlook towards intervention amongst intensivists that determined their responses. Indeed in a previous Canadian study, amongst intensivists the degree of aggressive treatment recommended was reflected less by the patients’ comorbidities than the physicians’ outlook towards aggressive treatments [10]. We also noted that intensivists were in general slightly better at outcome predictions, but this difference was not statistically significant.

Our study has several limitations. Given the modest sample size and single center nature of our study, our results are vulnerable to the same statistical liabilities to which small studies are prone. In addition, subtleties of a clinical situation can be difficult to convey through a clinical vignette and this may have influenced the responses. Finally, this study was conducted within a single university network and may not be generalizable to other centers. As such, a larger study to confirm these findings and to explore impact of individual characteristics should be performed.

Despite the limitations of our study, we believe that the results provide valuable insight into the dynamics of patient flow in an intensive care unit. It is well established that physician-related variability exists in end-of-life decision making in intensive care [14]. The large inter-individual variability found in our study shows that such variability is manifest as early as during triage and may have significant impact on the allocation of scarce resources. Indeed, such findings may be the result of a lack of fixed rules to guide ICU admissions. In our system, the decision to admit a patient to the ICU is left to each individual attending in charge of the unit on a particular day. Establishing triage consensus in the setting of scarce-resources has been identified as a priority by large organizations in the past such as, for example, Health Canada during the H1N1 pandemic [23]. Unfortunately, prior attempts at achieving such guidelines were limited by the uncertainty of patient outcomes [24]. Future research will need to integrate insight on critical care patients’ sequelae and outcome within a model of ICU triage consensus.

## Supporting Information

S1 Appendix (PDF)

S1 Ethics Approval (PDF)
