# Supplementary material for: The Perceived Likelihood of Outcome of Critical Care Patients and Its Impact on Triage Decisions: A Case-Based Survey of Intensivists and Internists in a Canadian, Quaternary Care Hospital Network
Source: PLoS One. 2016 Feb 12;11(2):e0149196. doi: 10.1371/journal.pone.0149196 (PMC4752246; doi:10.1371/journal.pone.0149196)
Supplement: S1 Appendix — (PDF) [file pone.0149196.s001.pdf]

Supplementary material

Appendix

1. Cases and questions distributed to participants

Survey
ILOVII
(ICU Likelihood of Outcome: Views of Internists and Intensivists)

Dear Participant,

You will be presented 5 cases. All of them were randomly selected and represent an ICU referral from a medical floor. After each case, you will be presented with a standard series of questions. Please make sure to answer all questions, even though they may sound similar. Please note there are no right or wrong answers; simply provide your decision and rationale as emulated by the case.

Section 1:

Case 1:

Age: 74
Sex: M
Living situation prior to hospital stay: Home
Number of days on medical floor prior to ICU consult: 1
Comorbidities: **-**Non-Small cell lung Cancer
-Chronic obstructive lung disease
On home oxygen
-Diabetes mellitus II
-Benign prostate hyperplasia
-Tremors NYD
Summary of events on medical floor: Admitted to the floor for a COPD exacerbation secondary to pneumonia. An arterial blood gas was performed because of an incidental low bicarbonate value found on a routine CHEM7. The ABG values were: 7.28/84/67/40
Reason for ICU consult: "Transfer for bipap"

Questions:

In the following questions: "High" is defined as greater than 70%

1. Do you think there is a high likelihood of this patient surviving their ICU admission?
□Yes □ No

2. Do you think there is a high likelihood of this patient surviving their hospital admission?

□Yes □ No

3. Do you think there is a high likelihood of this patient returning to his previous living situation?
□Yes □ No

4. Would you have indicated a different reason for ICU referral? If so, which one?
□Yes □ No □If yes only, please specify your ICU referral:

5. If you were the staff on the medical floor, would you have referred this patient to ICU?
□Yes □ No

5.a. Why?

6. If you were the staff in the ICU, would you have accepted the transfer?
□Yes□ No

6.a. Why?

7. How would you describe the patient's functional baseline prior to the acute event?
□ Good □ Poor

8. Notwithstanding of the patient's possible advanced directive, what level of care would you have recommended for this patient (see appendix A)?
□ Level 1 □ Level 2 □ Level 3

Case 2:

Age: 73
Sex: M
Living situation prior to hospital stay: Home
Number of days on medical floor prior to ICU consult: 2
Comorbidities: **-**Cirrhosis secondary to hepatitis B (MELD=18)
-Renal failure secondary to cryoglobulinemia (Baseline Cr=307)
-Retroperitoneal bleed on anticoagulation
-Pacemaker NYD
Summary of events on medical floor: Patient admitted from the ER with renal failure. On the floor, was found by the nurse pulseless and not breathing. Initial rhythm was paced with capture. A code blue was called, the patient was intubated and received epinephrine x2, atropine x1 and bicarb x1amp leading to return of spontaneous circulation. BP (systolic) augmented from 54mmHg to 230mmHg. Total downtime=6 minutes
Reason for ICU consult: "Post PEA arrest"

Questions:

In the following questions: "High" is defined as greater than 70%

1. Do you think there is a high likelihood of this patient surviving their ICU admission?
□Yes □ No

2. Do you think there is a high likelihood of this patient surviving their hospital admission?
□Yes □ No

3. Do you think there is a high likelihood of this patient returning to his previous living situation?
□Yes □ No

4. Would you have indicated a different reason for ICU referral? If so, which one?
□Yes □ No □If yes only, please specify your ICU referral:

5. If you were the staff on the medical floor, would you have referred this patient to ICU?
□Yes □ No
 5.a. Why?

6. If you were the staff in the ICU, would you have accepted the transfer?
□Yes□ No
 6.a. Why?

7. How would you describe the patient's functional baseline prior to the acute event?
□ Good □ Poor

8. Notwithstanding of the patient's possible advanced directive, what level of care would you have recommended for this patient (see appendix A)?
□ Level 1 □ Level 2 □ Level 3

Case 3:

Age: 84
Sex: M
Living situation prior to hospital stay: Home
Number of days on medical floor prior to ICU consult: 30
Comorbidities: **-**Coronary artery bypass graft
-Bioprosthetic aortic valve replacement
-Chronic kidney disease (baseline Cr=149)
-Prostate cancer
-Hypertension
-Hypothyroidism
-Dyslipidemia
-Arthritis
-Spinal stenosis
-?Chronic obstructive pulmonary disease

Summary of events on medical floor: Admitted with confusion, nausea, lethargy and urinary retention. Underwent placement of bilateral percutaneous nephrostomy 14 days later because of acute on chronic renal failure secondary to obstructive uropathy. Onset of c.diff diarrhea and became septic 2 weeks later.
Reason for ICU consult: "Septic shock"

Questions:

In the following questions: "High" is defined as greater than 70%
1. Do you think there is a high likelihood of this patient surviving their ICU admission?
□Yes □ No

2. Do you think there is a high likelihood of this patient surviving their hospital admission?
□Yes □ No

3. Do you think there is a high likelihood of this patient returning to his previous living situation?
□Yes □ No

4. Would you have indicated a different reason for ICU referral? If so, which one?
□Yes □ No □If yes only, please specify your ICU referral:

5. If you were the staff on the medical floor, would you have referred this patient to ICU?
□Yes □ No
5.a. Why?

6. If you were the staff in the ICU, would you have accepted the transfer?

□Yes□ No

6.a. Why?

7. How would you describe the patient's functional baseline prior to the acute event?

□ Good □ Poor

8. Notwithstanding of the patient's possible advanced directive, what level of care would you have recommended for this patient (see appendix A)?

□ Level 1 □ Level 2 □ Level 3

Case 4:

Age: **82**
Sex: M
Living situation prior to hospital stay: Home
Number of days on medical floor prior to ICU consult: 44
Comorbidities: **-**Lung cancer (right lower lobectomy 10 years prior)
-Chronic obstructive lung disease
-Coronary artery disease
-Hypertension

Summary of events on medical floor: The patient was admitted with abdominal pain and anemia investigated to be due to recurrent GIB secondary to dieulafoy lesions for which he had a prolonged course on the ward. 44 days after admission, his hemoglobin dropped to 58 for which he was transfused and ICU was consulted. The next day, he passed a large amount of melena but a g-scope is performed only the morning after. This revealed multiple actively bleeding duodenal ulcers. Pantoloc and octreotide are started while patient is still on the medical floor. The next day, he became hypotensive and short of breath with an elevation in his white blood cell count.

Questions:

In the following questions: "High" is defined as greater than 70%

1. Do you think there is a high likelihood of this patient surviving their ICU admission?

□Yes □ No

2. Do you think there is a high likelihood of this patient surviving their hospital admission?

□Yes □ No

3. Do you think there is a high likelihood of this patient returning to his previous living situation?

□Yes □ No

4. Would you have indicated a different reason for ICU referral? If so, which one?

□Yes □ No □If yes only, please specify your ICU referral:

5. If you were the staff on the medical floor, would you have referred this patient to ICU?
□Yes □ No

5.a. Why?

6. If you were the staff in the ICU, would you have accepted the transfer?
□Yes□ No

6.a. Why?

7. How would you describe the patient's functional baseline prior to the acute event?
□ Good □ Poor

8. Notwithstanding of the patient's possible advanced directive, what level of care would you have recommended for this patient (see appendix A)?
□ Level 1 □ Level 2 □ Level 3

Case 5

Age: **53**
Sex: F
Living situation prior to hospital stay: Home
Number of days on medical floor prior to ICU consult: 44
Comorbidities: -Diffuse large B cell lymphoma
-involving chest, abdomen and pelvis
-had 3 lines of chemo in the past 5 months with mild disease progression

Summary of events on medical floor: Admitted to the oncology floor for stem cell harvest. Received chemotherapt (cyclo-vp-16 on day2 and day9), then complicated by febrile neutropenia on day 14. She was started on Cipro and Vanco. On day 16, while still neutropenic, she becomes hypotensive ,tachycardic and febrile: BP 80/25 HR:127 and T:39.1. Responding transiently to fluid boluses with the BP improving to low 100s, however the mental status stable and urine output decreased.
Reason for ICU consult: "shock"

In the following questions: "High" is defined as greater than 70%

1. Do you think there is a high likelihood of this patient surviving their ICU admission?
□Yes □ No

2. Do you think there is a high likelihood of this patient surviving their hospital admission?
□Yes □ No

3. Do you think there is a high likelihood of this patient returning to his previous living situation?

□**Yes** □ **No**

4. Would you have indicated a different reason for ICU referral? If so, which one?
□**Yes** □ **No** □**If yes only, please specify your ICU referral:**

5. If you were the staff on the medical floor, would you have referred this patient to ICU?
□**Yes** □ **No**
    5.a. Why?

6. If you were the staff in the ICU, would you have accepted the transfer?
□**Yes**□ **No**
    6.a. Why?

7. How would you describe the patient's functional baseline prior to the acute event?
□ **Good** □ **Poor**

8. Notwithstanding of the patient's possible advanced directive, what level of care would you have recommended for this patient (see appendix A)?
□ **Level 1** □ **Level 2** □ **Level 3**

Section 2:

Please provide us with some information about yourself.

1. Please indicate your age

2. Please indicate your sex

3. How many weeks of ICU do you do during an academic year ?
□**0-2weeks** □**2-4 weeks** □**4-12 weeks** □**12-16 weeks** □**Greater than 16 weeks**

4. How many weeks of wards do you do during an academic year ?
□**0-2weeks** □**2-4 weeks** □**4-12 weeks** □**12-16 weeks** □**Greater than 16 weeks**

5. Please indicate the number of years you have been in practice as attending staff
□**0-5 years** □**5-10 years** □**10-15 years** □**15-20 years** □**Greater than 20 years**

Appendix A

Level of care*

1. Full care
2. Restriction on some technologies or aggressive modalities (please precise which ones)
3. No aggressive or surgical measures; medical treatment of reversible conditions.
4. Treatment aimed at comfort only.

*Inspired by McGill University Health Center Level of Intervention forms
