# Supplementary material for: The Perceived Likelihood of Outcome of Critical Care Patients and Its Impact on Triage Decisions: A Case-Based Survey of Intensivists and Internists in a Canadian, Quaternary Care Hospital Network
Source: PLoS One. 2016 Feb 12;11(2):e0149196. doi: 10.1371/journal.pone.0149196 (PMC4752246; doi:10.1371/journal.pone.0149196)
Supplement: S1 Ethics Approval — (PDF) [file pone.0149196.s002.pdf]

Supplementary material

Ethics approval:

Hôpital général juif
Jewish General Hospital

BUREAU D'ÉTHIQUE DE LA RECHERCHE
RESEARCH ETHICS OFFICE

Carolyn Ells, Ph.D.
Chair, Research Ethics Committee
Bureau / Room: A-925
Tel: 514-340-8222 x 2445
Fax: 514-340-7951
Email: carolyn.ells@mcgill.ca
Website : jgh.ca/rec

January 10, 2012

Drs. Dev Jayaraman and Joseph Dahine
Adult Critical Care
Jewish General Hospital

SUBJECT: **Retrospective Chart Review CR12-01 "Differences of perception between internists and intensivists with regards to likelihood of ICU outcome"**

Dear Drs. Jayaraman and Dahine,

Subsequent to the receipt and review of the above-mentioned project, please be advised that your request for permission to review medical charts at the Jewish General Hospital as part of your clinical research project is granted.

It is our understanding that the purpose of this chart review is to analyze the discordance in perceptions between two groups of specialists (i.e. intensivits and internists) pertaining to likelihood of ICU outcome based on multiple factors such as premorbid functional status, cormorbidities and reason for admission. This retrospective chart review will be done by Dr. Joseph Dahine. The data collected will be anonymized and kept on Dr. Dahine's computer for one year. No contact will be made with patients at any time during this retrospective chart review.

This approval is for the period of one year at which point you must request permission once again. Please contact Ms. Louise Tanguay, Director at the Medical Records Department of the Jewish General Hospital to arrange for consulting charts. She may be contacted at (514) 340-8222, ext. 8202.

Sincerely,

Carolyn Ells, Ph.D
Chair, Research Ethics Committee

Joseph Portnoy, MD
Director of Professional Services

CE/kb
CR12-01 chart review approval

CC: Ms. Louise Tanguay, Tel: 514-340-8222, ext. 8202
Director of Medical Records, Room C-110

Carmen Tapia – DPS A-142
